# Network Models for Assessing the Co‐occurrence Between Stuttering and ADHD

**DOI:** 10.1111/1460-6984.70320

**Published:** 2026-08-19

**Authors:** Fjorda Kazazi, Peter Howell

**Affiliations:** ^1^ Division of Psychology and Language Sciences University College London London UK

**Keywords:** ADHD, co‐occurrence, diversity, network models, phonological working memory, stuttering

## Abstract

**Background and Aims:**

Previous studies have indicated that people who stutter (PWS) and people with ADHD (PWADHD) show similar cognitive profiles, implying a link between the two neurodevelopmental profiles. This study examined the relationship between stuttering and ADHD and investigated the extent of this similarity using Network Models (NMs).

**Methods and Procedures:**

Neurotypical participants (people who did not stutter and did not have ADHD; *N* = 67), PWADHD (*N* = 79) and PWS (*N* = 33) were assessed for stuttering, ADHD traits, and phonological working memory (PWM). Lower PWM is associated with many conditions including stuttering and ADHD.

**Outcomes and Results:**

NM analysis revealed differences in cognitive networks (fluency, attention and PWM) between participant groups. The findings suggest partially different cognitive architectures across participant groups indicating that stuttering and ADHD do not share a common underlying mechanism. There were marked differences between participant groups in the way that traits of attention, stuttering, and PWM linked with each other which emphasises partially unique cognitive architecture of these participant groups. Higher PWM scores were associated with better attention in the neurotypical group and PWADHD but not PWS. Higher stuttering characteristics affected PWM in PWADHD and PWS, but the link was stronger in PWS. Whilst higher stuttering characteristics correlated positively with lower attention in PWADHD, the opposite was the case for PWS. PWM was the most important factor in all groups but the way it affected other cognitive processes differed between neurotypical participants, PWS and PWADHD.

**Conclusions and Implications:**

Overall NM structures were similar between the neurotypical group and PWADHD but they both differed from those of PWS. Findings argue against a shared underlying mechanism of attention, fluency and PWM in stuttering and ADHD and highlight the importance of including PWM assessments within a network‐based framework.

**WHAT THIS PAPER ADDS:**

*What is already known on this subject*

- Existing research indicates that people who stutter (PWS) and people with ADHD (PWADHD) exhibit overlapping traits in attention, speech fluency, and phonological working memory (PWM). Past studies have reported lower performance in attention, speech fluency and PWM in PWADHD and PWS as compared to neurotypical participants. This trait overlap has often been interpreted as co‐occurrence between the two profiles (stuttering and ADHD). However, existing literature has primarily relied on trait co‐occurrence and group‐level performance differences, without examining whether attention, fluency, and PWM interact in similar ways across the two profiles. As a result, it remains unclear whether these shared traits reflect common underlying mechanisms or distinct cognitive profiles that show similar behavioural outcomes.

*What this study adds to existing knowledge*

- The present study showed that although attention, fluency, and PWM differ between neurotypical participants versus PWS, and PWADHD, the way these abilities are interconnected differs between groups. Network analyses revealed partially distinct patterns of association, with the PWADHD network more closely resembling that of the neurotypical participants than that of PWS. Whilst PWM was a central component across all groups, its role within the network varied. In neurotypical participants and PWADHD, PWM was closely linked to attention, but this link was lost in PWS. These findings indicate that similar trait profiles do not necessarily imply the same cognitive architecture, and that overlapping traits in stuttering and ADHD can arise from different patterns of interaction among attention, fluency and PWM rather than from a shared underlying mechanism.

*What are the actual clinical implications of this study?*

- Our findings suggest that overlapping traits in attention, speech and PWM should not automatically be interpreted as evidence of a shared underlying profile in stuttering and ADHD. Instead, clinical evaluation should consider how cognitive processes interact within each profile. The results also highlight the value of including PWM assessment when examining attentional and fluency traits in both PWS and PWADHD, particularly using tools such as the UNWR. More broadly, network‐based approaches offer a promising framework for distinguishing between surface‐level trait overlap and fundamental differences in cognitive organisation, thereby supporting more precise, profile‐specific intervention strategies.

## 1 Introduction

Phonological working memory (PWM) is an important component which serves as a bridge between speech processing and attentional control (Baddeley 2012; Jacquemot and Scott 2006), making it relevant to neurodevelopmental profiles in which fluency and attention vary. Past research suggests that variations in attention occur in people who stutter (PWS; Alm and Risberg 2007; Donaher and Richels 2012; Druker et al. 2019; Healey and Reid 2003) as well as in people with ADHD (PWADHD; American Psychiatric Association, 2022; Chhabildas et al. 2001). Variations in speech fluency have been reported in PWADHD (Engelhardt et al. 2010; Lee et al. 2017), as well as in PWS (American Psychiatric Association 2022). PWM also varies in both conditions (Jacquemot and Scott 2006; Marchetta et al. 2008; Martinussen et al. 2005; Postma and Kolk 1993). Another similarity is that both are classified as neurodevelopmental disorders^1^ under the Diagnostic and Statistical Manual of Mental Disorders, 5th Edition (DSM‐5; American Psychiatric Association 2022).

Defining the co‐occurrence of stuttering and ADHD as those PWS who also report variations in attention leads to estimates between 4% and 26% (Arndt and Healey 2001; Riley and Riley 2000). Similarly, classifying the co‐occurrence of ADHD and stuttering as those PWADHD who stutter, leads to a rate of 18% (Biederman et al. 1993; Engelhardt et al. 2010; Jacobson et al. 2011; Tucha et al. 2005). Such overlap reported in stuttering and ADHD suggests a cognitive link between the two profiles. However, reports of shared traits such as these only show that PWS and PWADHD have similar surface characteristics. Such analyses fail to show: (1) whether attention and fluency variations correlate; and (2) whether the PWM processes that support speech and attention operate similarly or differs across neurodevelopmental profiles.

To address these points, PWS, PWADHD, were assessed on attention, fluency and PWM and compared to neurotypical participants to understand how these features connect with each other. To date, no study has examined whether features of attention and fluency are interconnected in a similar or different way across groups whose primary condition is either stuttering or ADHD and whether the PWM processes that support both features operate similarly or in a different way across groups. For instance, do PWM, attention and fluency correlate in the same way for PWADHD and PWS but in different ways for neurotypical participants as would be required to claim that ADHD and stuttering share a common cognitive mechanism that distinguishes them from neurotypical participants?

Accordingly, PWM and how it links with speech and attention is first reviewed, followed by a review of stuttering and ADHD and their shared features. Research on PWM in PWS and PWADHD is then discussed, followed by consideration of co‐occurrence of features. Evidence in stuttering research, shows that network science is valuable in demonstrating that communication processes can be modelled as interconnected features (Castro et al. 2017) and is therefore appropriate to assess the cognitive architecture of each condition and the connectivity of each feature. Traditional approaches such as the latent variable model (LVM) are compared to network models (NMs) outlining their limitations in revealing how features interconnect and the NM approach is introduced along with its pros and cons to compare the cognitive architecture of PWS and PWADHD.

### 1.1 Phonological Working Memory

The broad term ‘Working memory’ (WM) refers to a limited capacity mechanism that temporarily stores and processes information required to accomplish difficult cognitive tasks such as learning and problem‐solving (Cowan 2013). WM has four main components (Baddeley 2003): (1) central executive which is responsible for controlling actions and maintaining attention; (2) phonological loop which is responsible for managing linguistic and auditory information by rehearsing and refreshing that information (also known as PWM); (3) visuospatial sketch pad that allows visual information such as maps to be registered; and (4) the episodic connection which connects the phonological loop with the visuospatial sketch pad.

The phonological loop maintains verbal information over short intervals through a phonological store and a subvocal rehearsal process (Baddeley et al. 1998). It holds unfamiliar sound patterns temporarily to extract their phonological forms to create permanent phonological structures in the mental lexicon. PWM is closely tied to how speech is perceived and produced that operate together as part of two phonological buffers (Jacquemot and Scott 2006). Hence speech perception is part of the temporarily storage of phonological input and speech production is part of the subvocal rehearsal and inner articulation. These buffer capacities are important in everyday speech such as: (1) when listening, the system must retain phonological information across syllables to identify words and; (2) when speaking, the system must hold the phonological output while words are shaped. Jacquemot and Scott (2006) highlighted the importance of PWM in speech perception and production and suggested to integrate PWM into models to understand how phonological information is temporarily maintained during speech processing.

According to Baddeley's multicomponent model, temporary storage and attentional control are part of WM and therefore PWM and attention are linked (Baddeley 2012). PWM holds verbal information in a phonological store and subvocal rehearsal, while the central executive coordinates attentional control and PWM within a broader WM system. Camos and Barrouillet (2014), extended this account proposing two complementary systems. The first system (consistent with Baddeley 2012), once established, does not fully depend on attention and holds verbal information through subvocal rehearsal using language production processes. The second system (executive loop) requires attention and holds verbal information through attentional refreshing, which reactivates information in memory before it is lost. Hence, verbal information can be maintained through either rehearsal or attention‐based refreshing. In demanding tasks, attention can be occupied and executive‐loop maintenance declines, but the phonological‐loop can still support recall suggesting that both systems can operate independently or together and that people can choose between them depending on the task demands.

PWM is therefore important in speech‐related cognition as it enables the process of speech input by holding temporarily new information and by linking perception and production systems for new spoken forms. PWM also relies on attention since verbal information is maintained by the central executive through rehearsal or attentional refreshing. Hence, PWM links speech processing and attentional control, allowing variations of fluency, attention, and phonological information to be compared across neurodevelopmental profiles. Hence, the following sections review stuttering and ADHD separately (Sections 1.2. and 1.3. respectively), before reviewing research on PWM in PWS and PWADHD (Section 1.4).

### 1.2 Stuttering

According to DSM‐5 (American Psychiatric Association 2022), stuttering is a neurodevelopmental disorder that is characterised by disturbances in normal speech fluency not appropriate for the individual's age and communication abilities. Stuttering may continue over development. Stuttered speech is characterised by frequent and apparent occurrences of one (or more) of the following: (1) Repetitions of sound and syllables; (2) Consonant prolongation; (3) Mispronounced words (e.g., pauses within a word); (4) Blocking that is audible or silent; (5) Word replacements that avoid difficult words; (6) Excessive physical stress in the production of words; and (7) Whole monosyllabic‐word repetitions.

Approximately 1% of the adult population stutter and many children who stutter (CWS) do not continue to stutter before adulthood (Bloodstein, 1995). This results in the prevalence of stuttering at young ages being significantly higher than in adulthood. Estimates range from 4% to 5% of the population having stuttered at some point over early development (Andrews et al. 1964; Craig et al. 2002). An injury to the brain (such as stroke or trauma) can result in stuttering. However, this form of stuttering has been distinguished by DSM‐5 from developmental stuttering. Stuttering can lower a person's overall quality of life by causing them to have negative emotions about themselves related to their speech (Craig and Tran 2006) and limits employment prospects (Klein and Hood 2004). PWS may be more susceptible to bullying (Langevin and Hagler 2004) and have more difficulty establishing friendships with peers than children who do not stutter (CWNS; Davis et al. 2002). Boy‐to‐girl ratios range from around 2:1 for young children, to 5.33 males to each female for older children (Dworzynski et al. 2007).

ADHD traits have been reported in PWS in past studies. For example, Alm and Risberg (2007) compared 32 adults who stutter (AWS) with neurotypical participants and found that AWS reported significantly more childhood ADHD traits. They also found two subgroups within AWS: (1) individuals with higher childhood ADHD traits were more likely to have experienced neurological incidents prior to the onset of stuttering and; (2) those with lower childhood ADHD traits were more likely to have had a family history of stuttering. Donaher and Richels (2012) evaluated parent‐reported ADHD ratings in 36 CWS using the ADHD Rating Scale. They found that more than half of the sample (58%) met the criteria for professional referral for further evaluation of ADHD traits. The authors also found that CWS who had co‐occurring diagnoses (e.g., Tourette syndrome or anxiety) were more likely to show ADHD traits. Similarly, from parent reports of 185 CWS, Druker et al. (2019) found that about half showed higher ADHD traits. Additionally, CWS who exhibited ADHD traits required additional therapy sessions for successful fluency outcomes compared to those without ADHD traits. One explanation for this is that the therapy outcomes depend on how well PWS utilise fluency techniques and those with ADHD traits may have more difficulties sustaining attention and controlling impulsive behaviours (Healey & Reid 2003).

Overall research suggests that stuttering may be heterogeneous and some individuals who stutter exhibit ADHD traits and this is reported more frequently in children. Such findings suggest that ADHD traits can be exhibited in PWS, and it is important that clinical assessments of PWS include further evaluations of ADHD.

### 1.3 ADHD

Attention deficit hyperactivity disorder (ADHD) is a neurodevelopmental disorder characterised by a persistent combination of inattentive, impulsive, and hyperactive behaviours (American Psychiatric Association, 2022; World Health Organization 1992). Inattention and disorganisation refer to reduced ability to stay on task and have listening difficulties that are not in accordance with one's age or developmental level (American Psychiatric Association 2022; Kofler et al. 2008). Inattentive behaviour is linked to various underlying cognitive processes. Hence PWADHD may struggle with executive function and memory assessments as well as attention. Overactivity, fidgeting, difficulty in staying seated, intruding into other people's activities, and inability to wait are all indicators of hyperactivity‐impulsivity. An ADHD diagnosis is considered when inattentive or hyperactivity traits exceed what is generally exhibited in people of equivalent mental age. PWADHD can have a presentation predominantly on attention, hyperactivity‐impulsivity or a combined presentation. ADHD begins in childhood. The requirement that five or more symptoms of inattention and five or more symptoms of hyperactivity must be evident before the age of 12 years and should persist for at least 6 months emphasises the clinical presence in childhood (American Psychiatric Association 2022). ADHD symptoms must impact academic and social performance and should be persistent in at least two settings (such as home, school or in different social activities). ADHD frequently continues throughout adulthood, affecting various aspects of life such as social, intellectual, and occupational.

Meta‐analyses studies suggest that ADHD affects approximately 5%–7% of children and adolescents across countries (Polanczyk et al. 2007; Willcutt 2012) while the rate for adults is about 2.5% (Simon et al. 2009). In the general population, males are more likely than females to have ADHD, with a ratio of approximately 3:1 in children (Biederman et al. 2002) and 1.6:1 in adults (Stibbe et al. 2020). Females are more prone than males to have inattentive traits (Biederman et al. 2004) or to have the combined ADHD subtype (Simpson 1969) whilst males have more hyperactive/impulsive traits (Ramtekkar et al. 2010). The gender ratio changes over age leading to more females than males being diagnosed with ADHD. This change may be because hyperactive traits in males decline with age. Alternatively, for females, it is possible that some unrecognized form of adult‐onset of ADHD occurs such that focusing only on childhood onset statistics misrepresents gender imbalances, but this remains to be studied. Research may benefit from the use of non‐clinical populations to avoid referral bias (Levy et al. 1997). Hence, in the present study PWADHD in the ADHD group had three different backgrounds: (1) ADHD clinical diagnosis; (2) self‐reported ADHD diagnosis; (3) participants reported traits of inattentive and hyperactivity in line with the DSM‐5 criteria for having a presentation of ADHD. People with backgrounds (2) and (3) are combined as the non‐clinical group.

Fluency characteristics have been reported in PWADHD. Engelhardt et al. (2010) assessed speech fluency characteristics in adolescents and adults with ADHD during a sentence production task. Compared with neurotypical participants, PWADHD (those with combined ADHD subtype) produced more stuttering‐like characteristics, especially when sentence planning was more difficult. The authors argued that fluent speech may depend on inhibitory control, and a weaker inhibition control may make it difficult to prevent and correct speech errors during production. Lee et al. (2017) assessed speech fluency characteristics in 15 children with ADHD (CWADHD) and compared them to 15 age‐matched neurotypical children during reading, story retelling, and picture description tasks. CWADHD produced significantly more stuttering‐like speech characteristics across all tasks. Elevated stuttering‐like speech characteristics were observed mostly during the story retelling task which requires greater attention. Authors concluded that when attentional demands were higher, CWADHD exhibited more variable fluency.

Findings suggest that PWADHD exhibit variations in fluency in tasks with higher demands of attention, planning, and inhibitory control. As discussed in Section 1.1., PWM is linked to attention and verbal processing, and executive control supports both PWM and speech. Hence, differences in maintaining phonological information during speech in PWADHD compared to neurotypical participants, may explain stuttering‐like characteristics.

### 1.4 Phonological Working Memory in Stuttering and ADHD

Conditions in which communication is affected, have mainly been linked to executive functioning (EF) and PWM. It has been suggested that PWM is involved in planning and producing speech since these processes are involved in accessing linguistic information to build articulatory plans (Gathercole and Baddeley 1993; Jacquemot and Scott 2006). To create articulatory plans during speech planning, phonological information is retrieved from memory and processed through phonological encoding which relies mostly on phonological loop (Levelt 1989).

Fluent speech production can be defined as the precise articulation of phonemes that comprise spoken words. Fluency requires phonological knowledge about speech sounds as well as the capacity to synchronise articulator movements (jaw, tongue, and lips) with breathing and vocalisation. It has been suggested fluency variations may be explained as differences in how linguistic planning and motor execution processes are coordinated (Howell 2004). According to Howell's (2004) EXPLAN model, such disruptions may occur when there is temporal asynchrony between planning (PLAN) and execution (EX) processes. For instance, when speech is generated quickly, when the word is phonologically complex, when an easy word precedes a more complex word, or when speech is made before the next word is planned.

Daneman (1991) reported a link between fluency and PWM in fluent speakers. Subsequently, phonological encoding has been assessed in PWS (Melnick et al. 2003). One hypothesis is that phonological encoding is affected in PWS and this leads to errors in speech planning that manifest as stutters when PWS deal with these challenges (Postma and Kolk 1993) thus implicating PWM. Alternatively, the EXPLAN model, outlined above, proposes that fluency variations may reflect timing and coordination between planning and execution (Howell 2004) rather than phonological encoding difficulties alone. Kinematic evidence suggests that increasing speech demands can heighten instability in inter‐articulatory coordination of PWS. In Kleinow and Smith (2000), eight AWS and eight neurotypical participants were asked to produce a target phrase (e.g., “Buy Bobby a puppy”) either alone or embedded in longer or more complex utterances while lower lip movements were recorded. Authored measured the spatiotemporal index (STI) for the movement stability. Results showed that AWS exhibited higher STI values across conditions, indicating that during speech production, AWS had greater variability in speech motor output. Increasing only the complexity of syntax, reduced stability in AWS as compared to neurotypical participants whereas increasing only the length did not show any significant differences. It was concluded that the speech motor systems of AWS may be especially affected by the linguistic demands of producing more complex utterances. More recent work (Wiltshire et al. 2021) has extended these findings by using real‐time vocal tract magnetic resonance imaging (MRI). Twenty‐eight AWS and 20 neurotypical participants were assessed during simple and complex pseudowords that varied in syllable length and phonological complexity. Findings showed that AWS exhibited greater variability in movements of the lips, tongue, and velum and produced longer durations of the movements during multisyllabic pseudowords particularly as phonological complexity increased. Wiltshire et al. (2021) work has added that PWS show more variability in articulatory control during fluent speech production.

As outlined in Section 1.1, PWM involves temporary phonological storage, subvocal rehearsal, speech perception and production, and coordination with attentional/executive control (Baddeley 2012; Camos and Barrouillet 2014; Jacquemot and Scott 2006), all of which are relevant to variations of fluency, attention and PWM in PWS and PWADHD. Evidence suggests that non‐word repetition (NWR) tasks provide a direct behavioural index of these processes and are particularly sensitive to differences in phonological memory skill (Gathercole et al. 1994). However, NWR tasks measure more than temporary phonological storage. Successful repetitions depend on speech perception, phonological encoding, temporary storage and rehearsal, and planning and execution of articulatory output (Coady and Evans 2008; Gathercole et al. 1994). In a nonword repetition task, Elsherif et al. (2021) found that AWS differed significantly from neurotypical adults, with variations attributed to phonological encoding and subvocal rehearsal rather than passive storage alone (Byrd et al. 2015; Pelczarski and Yaruss 2016). Compared with other verbal tasks with a phonological memory component that rely on familiar lexical items (e.g., auditory digit span) or involve lower speech output demands (e.g., visuospatial WM), in NWR tasks, participants are required to encode, maintain and produce unfamiliar phonological forms (Gathercole et al. 1994). NWR tasks provide an appropriate measure of the PWM processes since accurate repetitions depend on the overlap of phonological (hear and hold unfamiliar sounds), attentional/executive (maintain focus and keep information active) and speech‐motor processes (plan and execute speech output) which have been implicated in stuttering and ADHD. Therefore, NWR tasks have been utilised to assess performance in perceptual, memory, and speech‐output processes in stuttering (Coady and Evans 2008) and ADHD (Bolden et al. 2012) particularly using multisyllabic non‐words that impose greater PWM demands (Norrelgen et al. 1999).

Several studies have compared NWR performance in PWS and neurotypical participants with mixed results. Some studies (Anderson and Wagovich 2010; Bakhtiar et al. 2007; Byrd et al. 2015; Hakim and Ratner 2004; Tichenor et al. 2022) reported lower accuracy in PWS compared to neurotypical participants. Anderson and Wagovich (2010) conducted a picture naming task where speech reaction time (SRT) was measured as well as a NWR task in child groups. No difference was observed in the speed of naming the picture, but CWS performed significantly lower in the PWM nonword task compared to CWNS. Also, Hakim and Ratner (2004) investigated children in a NWR task (Gathercole et al. 1994). In the NWR task, CWS had lower accuracy and produced more phoneme errors than CWNS. Similar results were obtained with CWS by Bakhtiar et al. (2007), with adults by Tichenor et al. (2022) and with adults and children by Byrd et al. (2015).

However, other studies did not find a group difference and report comparable accuracy between groups, particularly when PWS have typical speech and language or when tasks utilise shorter and phonologically simpler words (Gerwin et al. 2022; Smith et al. 2012). In Gerwin et al. (2022) poorer NWR performance was driven by co‐occurring speech and language disorder while CWS with typical speech and language did not differ from CWNS. Similarly, Smith et al. (2012) did not find significant differences in accuracy in the NWR task in CWS compared to neurotypical participants, despite the higher variability observed in oral motor coordination during accurate repetitions.

Findings are consistent with evidence that PWS often have intact phonological storage and rehearsal abilities (Pelczarski and Yaruss 2016; Smith et al. 2012) but may show neural differences in rehearsal and executive control rather than in load‐dependent storage (Yang et al. 2019). Hence, simple NWR tasks that assess storage demands might not reveal significant differences (Gerwin et al. 2022). Instead, when NWR tasks place greater demands of phonological information or the complexity of phonological representations, group differences are more likely to emerge (Gerwin et al. 2022; Pelczarski and Yaruss 2016; Yang et al. 2019). Group differences are often revealed when syllable length or phonetic demands are increased. For example, Coalson et al. (2019) found that AWS were less accurate than neurotypical participants only for non‐words with greater segmental complexity, but no differences were found for simple or moderate forms. Similarly, Sasisekaran et al. (2019) reported that as complexity increase, inter‐articulatory coordination of CWS for 3‐syllable non‐words and slower movements was significantly reduced. In another study, Sasisekaran and Weathers (2019) showed that CWS exhibited more stuttering‐like characteristics during longer non‐words and made fewer phonological revisions compared to neurotypical participants. Findings suggest that group differences may be due to higher demands for encoding and monitoring demands rather than storage differences alone. Kinematic studies reveal similar results. Smith et al. (2010) assessed whether increasing length and phonological complexity of non‐words in the NWR task, affected speech motor control in AWS. Seventeen AWS and 17 neurotypical participants repeated non‐words that varied in length and phonological complexity while lip movements were recorded. There were no differences in accuracy to repeat the non‐words, but as non‐word length and complexity increased, AWS showed less consistent coordination among the upper lip, lower lip, and jaw. Additionally, AWS were slower in producing the non‐words and became more consistent across later trials. Smith et al. (2010) concluded that despite the similar accuracies observed between groups, the NWR task revealed crucial differences in speech motor dynamics during speech. Overall, findings suggest that PWM differences in stuttering are more prevalent when the cognitive‐motor load of encoding, rehearsal, and speech planning and execution is increased. More complex NWR tasks that tax these processes are more likely to reveal group differences compared to short traditional NWR tasks.

Lower WM performance has also been reported in PWADHD in one study which examined PWM using a NWR task (Norrelgen et al. 2007). They sub‐divided CWADHD into two types: CWADHD who had ADHD alone or ADHD and a developmental coordination disorder (CWADHD+) and compared performance of each group separately against neurotypical children. The CWADHD+ had lower PWM scores than other groups.

Other studies on PWM and ADHD have focussed on visual encoding in WM rather than PWM and, in some cases, have assessed PWM in tasks other than NWR where results may possibly be mediated by linguistic factors. As an example of the former, Raiker et al. (2017) examined visual encoding, visual to phonological conversion and measures speech response output in CWADHD and neurotypical group. As an example of the latter, Kofler et al. (2011) evaluated the effects of central executive, phonological WM, and visuospatial WM on social difficulties as reported by parents and teachers of CWADHD and neurotypical participants. For the PWM task, children were given a sequence of randomly arranged numbers and a capital letter, and they were asked to recall the numbers in ascending order from lowest to biggest while also saying the letter last (e.g., the stimulus 2 A 5 1 would be correct if a participant responded 1 2 5 A).

In sum, there is ample evidence that PWS show lower performance in NWR tasks than neurotypical participants and, similarly for PWADHD although there is more limited evidence in this case. Such evidence suggests that PWM is affected in PWADHD as well as in PWS. Hence, it is appropriate to investigate PWM performance in PWS and PWADHD in NWR tasks. However, NWR tasks used in investigations to date prioritise the language for which they were designed, which may limit their applicability when presented to people who speak other languages. For example, Greek‐speaking people who also speak English did better on Greek than English NWR tests (Masoura and Gathercole 1999). The universal NWR (UNWR) of Howell et al. (2017) partly addresses this limitation because it is applicable to 20 commonly spoken languages in the UK. Additionally, the syllable length of stimuli in UNWR increases across set sizes and the task applies multisyllable construction rules that place increasing demands on dynamic processing beyond passive phonological storage. Hence UNWR can be used to identify variations in fluency in linguistically diverse samples. The UNWR task (Howell et al. 2017) was employed in this study to understand how PWM relates to fluency and attention in PWS, PWADHD, and neurotypical participants.

The above literature suggests a possible link between stuttering and ADHD since they share core traits characteristic of the other condition (attention and fluency), are subject to similar environmental factors and demographic biases. As discussed in this section, PWM is also affected in both conditions. Consistent with this overlap, estimates of the co‐occurrence of stuttering and attention difficulties among people who stutter (PWS) range from 4% to 26% (Arndt and Healey 2001; Riley and Riley 2000), whereas estimates of stuttering among people with ADHD are approximately 18% (Biederman et al. 1993; Engelhardt et al. 2010; Jacobson et al. 2011; Tucha et al. 2005). However, shared surface traits do not necessarily reflect a shared underlying mechanism and overlapping features does not reveal how attention, fluency and PWM are organised within each condition. To date, no research has assessed how PWM links to fluency and attention or whether these features link in the same or different ways across stuttering and ADHD. To this end, the concept of co‐occurrence and the network models (NMs; Cramer et al. 2010) adopted here are described in the following sections.

### 1.5 Co‐occurrence

*Co‐occurrence* is the presence of two (or more) conditions simultaneously in an individual (Angold et al. 1999). It is important to determine multiple mental condition occurrence in one person since, when they are present, they pose higher demands for professional treatment, more difficulty coping with ordinary life, and a higher rate of suicide (Albert et al. 2008). Approximately 45% of people who meet diagnostic criteria for a given mental condition, receive additional diagnoses for other conditions (Kessler et al. 2005). Gadermann et al. (2012) suggested that this figure could be as high as 73.8%–98.2% if chronic physical disorders are included as co‐occurring conditions too. The assessment in these reports (Gadermann et al. 2012; Kessler et al. 2005) involved a replication of a US National Comorbidity Survey Replication (NCS‐R^2^). In NCS‐R, disorders from Diagnostic and Statistical Manual of Mental Disorders, 4th Edition (DSM‐4) were correlated with each other. In the adult psychiatric literature, there is a growing interest in co‐occurrence based on claims such as these and, to this end, the NCS‐R was developed (Angold et al. 1999).

As outlined above, reduced fluency and lower PWM can both be present in PWS and in PWADHD suggesting a link between the two conditions. However, no research has used statistical approaches to assess the relationship between these measures, for example, whether they are affected in the same or different ways across groups of participants where the primary condition is either stuttering or ADHD. As well as asking whether attention and fluency are affected in PWS, PWADHD and neurotypical participants, we also ask whether PWM and its connections with attention and fluency operate in the same way across groups. NM is used for this purpose, and details of this approach appear next.

### 1.6 Network Model

Conditions are viewed as clusters of directly related features in a “network perspective” (Borsboom and Cramer 2013; Cramer et al. 2010; Epskamp et al. 2017a; Epskamp et al. 2017b). To illustrate, Fried et al. (2016) used NM to investigate the relationships among features of depression. They reported that the essential features in depression were recognised by the networks and provided information on which features may be targeted during therapies. The network method assumes that features can interact with one another and hence be causally linked (Cramer et al. 2010) and is generally used to find and analyse statistical relationship patterns in multivariate psychological data (Borsboom et al. 2021).

**FIGURE 1:**
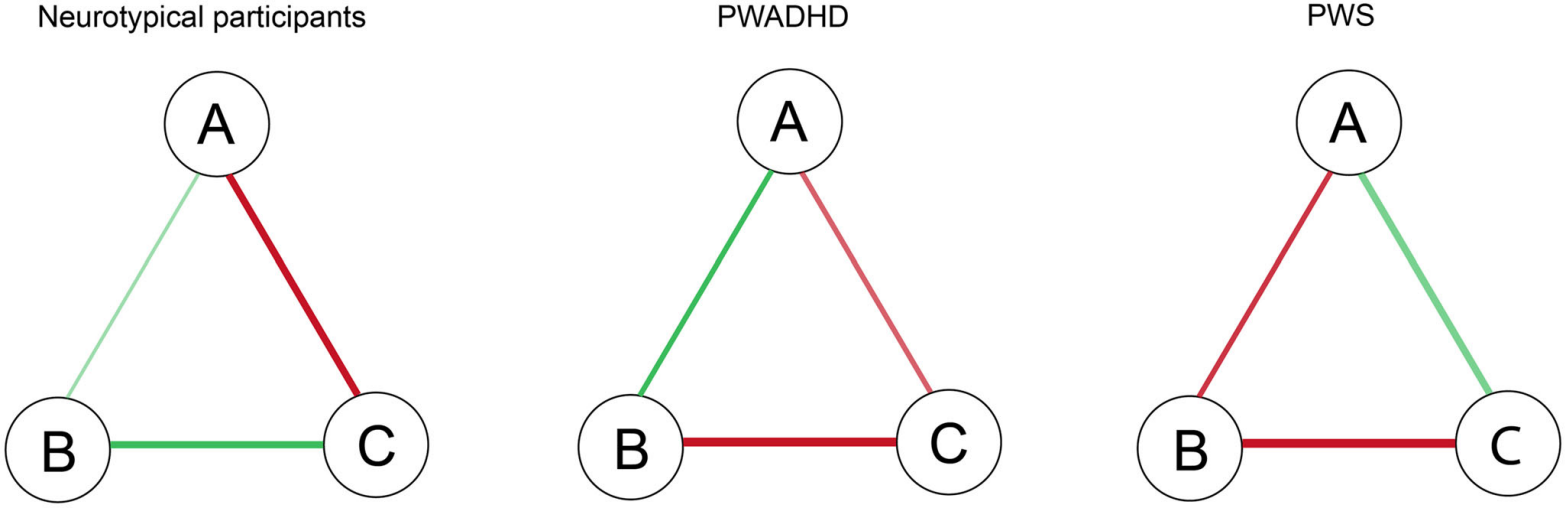
Examples of NMs visualised for neurotypical participants (left NM), PWADHD (middle NM), and PWS (right NM) for features A: Attention, B: Fluency, C: Phonological Working Memory. Green lines are positive connections and red lines negative connections. The thickness and colour intensity of the edges reflect the strength (weight) of the association, with thicker and more saturated edges indicating stronger relationships and thinner, lighter edges indicating weaker relationships.

Networks involve nodes and edges. Nodes represent the features (in circles) in the NM whereas edges are the lines that connect the nodes. The network's edges that link two nodes together, may or may not be directed (visualised with a line or an arrow) positively or negatively (green for positive connections; red for negative connections in Figure 1) and may or may not be weighted for their strength (edges can have a value or no value). For example, losing a family member (external factor) can activate a feature in the network (such as depression) which in turn can activate neighbouring features such as anxiety, insomnia and eating conditions. After a network has been built and nodes and connections have been established, the topology of the network can be examined using network science descriptive tools for the position of specific nodes inside the network. Network techniques are useful tools for analysing multivariate data and can be constructed for various purposes. They are particularly useful when no prior knowledge about the relationship among the variables is known, allowing researchers to investigate the structure of high‐dimensional data. Network representations also provide excellent visualisations of statistical association patterns and can be used to effectively explain multivariate patterns of reliance. Finally, because network models capture the statistical structure of the data, they can be used to construct causal hypotheses (Borsboom et al. 2021).

Network models can help us understand whether an individual who has met the DSM criteria for one primary condition (feature A, attention) shows features of another, secondary, condition (feature B, stuttering) and how these compare in individuals with primary and secondary conditions reversed. Furthermore, if a further feature (C, PWM) exists that is affected when either primary condition occurs, its relation to features A and B can be determined and patterns compared across conditions. For example, Figure 1 plots some network relationships across features A‐C for neurotypical participants, PWADHD and PWS. If only features A and B were obtained, the valences for the neurotypical group and PWADHD would be the same but different from PWS. However, the PWM features reveal differences in the way PWM works in the network across all participant groups.

Generally speaking, mental conditions represent an interconnected web of features (here, attention, fluency and PWM represented as nodes) and according to network theorists, the conditions are emergent phenomena that originate from direct interactions amongst their features, rather than underlying factors that cause them to appear (Jones et al. 2019). Mental conditions often have many features in common (shown in NM as nodes). For example, features of obsessive‐compulsive disorder (OCD), are associated with guilt, which in turn is a cause of depression (Kim et al. 2011). Certain features of one condition may increase the likelihood of others, leading to diagnostic co‐occurrence. Castro et al. (2017) applied network science to stuttering by reanalysing lexical decision data in AWS and neurotypical participants. A phonological network of the mental lexicon was constructed, with nodes representing words and edges representing connections between phonologically similar words. Findings showed that network measures such as degree/neighbourhood density and closeness centrality, predicted lexical decision reaction times beyond traditional psycholinguistic variables (e.g., word length and frequency), suggesting that modelling relationships between interconnected words rather than examining words in isolation, provides a better approach to understanding phonological processing. Network effects were similar across groups with a similar network structure observed in AWS and neurotypical participants suggesting comparable lexical‐network structure across groups. Although Castro et al. (2017) focused solely on psycholinguistic phonological network of the mental lexicon rather than cognitive‐feature networks, findings demonstrated the value of network science for understanding communication processes in stuttering as interconnected rather than isolated features. Extending this network approach to examining the interconnection of cognitive features (attention, fluency, and PWM) through NMs has not been conducted for this purpose to date. This extension is consistent with literature that PWM sits at the intersection of phonological, attentional/executive, and speech‐motor processes in both stuttering and ADHD (Sections 1.1. and 1.4.), with multifactorial accounts of stuttering in which linguistic and motor demands interact, and with accounts of ADHD in which attentional/executive control supports verbal maintenance and fluent speech under greater demand. Next, we introduce details about the pros and cons for NM based on work from other conditions.

#### 1.6.1 Pros and Cons of NM Relative to Other Approaches

The network account of co‐occurrence derives strong support from work on clinical treatments. Cramer et al. (2010) claimed that NM reflects the processes of the more successful therapeutic treatments for mental conditions which emphasises the importance of relationships among interconnected features. For example, Cognitive Behavioural Therapy (CBT; Beck 1979), treatment focuses on the impact of cognition on features e.g., how ruminating might lead to a spiral of irrational thoughts and subsequent actions. Furthermore, treatments utilising the LVM approach to co‐occurrence, which does not involve networks, have yielded contentious results. The LVM approach to mental disorders embodies a traditional view of co‐occurrence. Co‐occurrence in this context, is regarded as a relationship between multiple latent variables or due to a direct connection between two latent variables where the latent variables represent mental conditions (Neale and Kendler 1995). Some researchers go even further, hypothesising that a direct relationship between two latent variables signifies the presence of a “super disorder”. For instance, Barlow et al. (2004) described models in which the super disorder “negative affect” causes a number of mental conditions (such as depression). In this framework, negative affect is thought to trigger the observable features for the various conditions. Cramer et al. (2010) further noted that, under such LVM, associations between different features are often interpreted merely as covariance resulting from a common underlying cause rather than direct interactions between the features themselves. Historically, the assumptions behind such models have been the basis for diagnosis of mental disorders, in that disorders are seen as separate entities with unitary causes and definitive symptoms that can be used to identify the disorder a person has (e.g., 5th ed.; DSM‐5; American Psychiatric Association, 2022).

Cramer et al. (2010) suggested that the LVM approach to co‐occurrence research has significant flaws, and NM avoids these contentious concerns. For example, antidepressants do not work for every patient (Cipriani et al. 2009), indicating that the latent variable assumed for depression (e.g., serotonin imbalances) does not explain the condition for all patients. Thus, not only might the NM provide a better explanation for the causes of mental conditions, but it may also have positive implications for developing new treatment strategies. Cramer et al. (2010) pointed out that clinical practice has commonly taken a network‐style view of treatment (e.g., administering a range of treatments besides antidepressants to patients). Thus, the NM may advocate more of a psychometric rather than an applied change in research perspectives.

NM has strong foundations in genetic and neural research. Thus, Davis and Plomin (2010) noted that genetic influences on psychological phenotypes (i.e., behaviour) can be well represented in a network approach due to similarities in implicated genetic factors between conditions and features. Furthermore, a network approach is highly reflective of the anatomical and functional properties of the brain; brain imaging studies are progressively and consistently revealing the highly complex and interconnected nature of brain structure, termed the ‘connectome’ (e.g., Pessoa 2017). It seems likely, then, that co‐occurrence would function similarly in that traits correlate due to direct biological connections, such as shared genetic influences or neural networks.

Whilst NM is appropriate for characterising how fluency, attention and PWM relate within stuttering and ADHD, NM has not been used for this purpose to date. Hence this study uses NM to explore whether these overlapping features operate in the same or different way across PWS, PWADHD and neurotypical participants.

### 1.7 The Current Study

The literature suggests that fluency, attention and PWM are frequently affected in PWS or PWADHD. Conventionally, the presence of these shared features has been considered sufficient to designate the two conditions as co‐occurring. However, shared features do not necessarily imply a shared underlying mechanism and simply showing overlapping traits cannot reveal how features are organised within each condition. Hence, to determine the connectivity within the cognitive architecture of these conditions, the strength and direction of the relationship between these features should be assessed in a different approach using NMs. This should provide further support for how PWS and PWADHD differ from one another and from neurotypical participants beyond what shared features alone can indicate.

The main purpose of this study was to understand the attentional and fluency abilities of neurotypical participants, PWS and PWADHD using NM and to establish how these patterns are affected when PWM is included in the model. Attentional abilities were measured using the Adult ADHD Self‐Report Scale Screener (ASRS; Ustun et al. 2017), fluency using the Stuttering Severity Instrument, version 3 (SSI‐3; Riley 1994), and PWM using the UNWR test (Howell et al. 2017). Building on network approaches to phonological processing in stuttering (Castro et al. 2017), UNWR was selected as a measure of PWM since NWR tasks tap into the overlap of phonological encoding, attentional/executive and speech‐motor planning processes implicated in both stuttering and ADHD while also addressing the limitation of language bias and neighbourhood‐density confounds (see Sections 1.1. and 1.4.). Since the NMs implemented in the present study assess how PWM relates to attention and fluency, UNWR was therefore better suited to test the intersections of these features across groups than tasks that tap only into storage or involve lower speech‐output demands with familiar lexical input. Accordingly, UNWR allowed to test the following hypotheses regarding network connectivity. H1 was that the way ASRS, SSI‐3, UNWR measures are connected to each other in NM should differ between participant groups. H2 was that neurotypical participants would show high UNWR scores (better PWM abilities) and this should be negatively associated with low ASRS scores (better attention). H3 examined whether UNWR scores correlated with ASRS scores in PWS and PWADHD, but the direction (positive or negative) was not specified a priori. Networks were also compared for significance as well as connectivity patterns. Given that stuttering and ADHD are both classified as neurodevelopmental disorders in the DSM‐5 (American Psychiatric Association 2022), marked differences sufficient for significance may be difficult to achieve.

## 2 Methods

Participants were compensated for participating in the study and travelling expenses were reimbursed. As part of the university‐approved research programme, all assessments were conducted in English and required participants to have conversational or fluent English. Under the university's research ethics procedures, participation required that participants understand study materials (participant information sheet) and provide informed consent in English before testing.

### 2.1 Participants

Sixty‐seven neurotypical participants (people who did not stutter and did not have ADHD; Mean age: 24.25, SD: 6.709, 42 Females, 25 Males), seventy‐nine PWADHD (Mean age: 24.66, SD: 5.154, 35 Females, 44 Males) and thirty‐three PWS (Mean age: 34.18, SD: 11.796, 14 Females, 19 Males) were included in this study. Neurotypical participants were recruited from the university's SONA participant pool and none self‐reported stuttering, ADHD or another neurodevelopmental condition. PWADHD were recruited from online ADHD groups and forums. PWS were recruited from online self‐help groups in London and Manchester. Participants were grouped by primary condition (stuttering or ADHD) and co‐occurrence was not formally assessed. Hence no dual‐diagnosis subgroup was defined and participants with overlapping traits were retained. Overlapping traits were instead captured continuously across the three measures in the networks. Cross‐trait overlap analysis showed that while the overlapping traits were present, the subgroup sizes were limited for stable network estimations (see Section 3.). Because ASRS, SSI‐3 and UNWR were administered across several previously conducted studies, the sample size was not determined a priori. A sensitivity power analysis was conducted using the pwr package (v1.3.0) in R (v4.1.3), which implements the same analytic procedures as G*Power (Faul et al. 2007) and the following report is based on the guidelines from Lakens (2022). With *α* = 0.05 and 80% power, the design was sensitive to a between‐group effect of Cohen's *f* = 0.23 for the omnibus one‐way ANOVA across the three groups (*f* = 0.25 when adjusted for group imbalance using the harmonic‐mean group size, *n* = 51.8). For pairwise comparisons, the minimum detectable effects were *d* = 0.47 for neurotypical participants compared to PWADHD, *d* = 0.60 for neurotypical participants compared to PWS, and *d* = 0.59 for PWADHD compared to PWS. The observed group differences on all three measures were large (*ω* ^2^ = 0.18–0.55; corresponding to Cohen's *f* = 0.46–1.11; see Table 1) and thus well above the minimum effect the design was sensitive to (*f* = 0.23), indicating that the study was clearly able to detect the effects present in the data. Comparisons involving PWS (*N* = 33) could detect medium‐to‐large effects (*d* ≈ 0.59) while the detection of smaller may have been limited since power for pairwise contrasts relies primarily on the smallest group.

**TABLE 1:** Descriptive Statistics for ASRS, SSI‐3, UNWR.

	Neurotypical participants			PWADHD			PWS			Omega squared
	*M*	SD	*N*	*M*	SD	*N*	*M*	SD	*N*	
ASRS	9.95	3.614	66	16.84	2.821	79	13.72	2.667	32	0.495
SSI‐3	5.11	3.750	66	6.15	4.808	68	20.00	7.479	32	0.554
UNWR	22.12	5.973	67	17.04	5.190	77	15.94	5.750	33	0.175

*Note*: Omega squared represents the effect size: ω2 ≈ 0.01 is small effect; ω2 ≈ 0.06 is medium effect; ω2 ≈ 0.14 is a large effect.

### 2.2 Design

Participants conducted different experiments after the data used in the current study were collected. Before every experiment, participants completed the ASRS questionnaire, read two short paragraphs from the SSI‐3 manual and conducted the UNWR task. The order of the three tasks was counterbalanced. The dependent variables were ASRS, SSI‐3, UNWR and the between‐participants factor was the participants' group.

### 2.3 Materials

The ASRS questionnaire (Ustun et al. 2017) has six questions for participants to answer from never to very often. The questions are then scored based on the ASRS scale. The total ASRS score (dependent variable) was the sum of all scores achieved from all the questions. A total score ranged between 0 and 25 where higher scores indicated more ADHD traits. For the SSI‐3 measure (SSI‐3; Riley 1994), a video for each participant was analysed based on the frequency of stuttering (repetitions, prolongations, word breaks), duration of stutters (both frequency and duration measures were scaled) and physical concomitants (e.g., jaw tension). The total score (dependent variable) was the sum of all these variables, and the percentile and severity of stuttering were determined from the total score. A higher total score indicated more severe stuttering. UNWR (Howell et al. 2017) was implemented as a web application, and for each set of non‐words, participants were scored on how many words they repeated correctly. A total score on UNWR (maximum possible score was 32) was the dependent variable. A higher UNWR score indicated better PWM. NWR tasks have been developed to assess unfamiliar phonological forms rather than lexical items, and UNWR (Howell et al. 2017) was selected to reduce psycholinguistic confounds observed in standard NWR tasks. Stimuli were constructed from shared phonotactic templates with consonant and vowel inventories across commonly spoken UK languages studied and were verified to ensure that items were not real words in the target languages. From the word‐likeness analysis, it was concluded that the UNWR items showed substantially lower neighbourhood density than the Children Test of NWR (CNRep; Gathercole et al. 1994), subsequently reducing similarity on a person's lexical knowledge and word‐likeness effects. Scoring is based on consonants which further reduces the influence of accents and lexical effects of vowel pronunciations. Stimuli increase from two to five syllables across four set sizes which increase in length, with progression to the next set based on accurate repetition. Stimuli follow the same phonotactic and concatenation rules. Hence, UNWR places greater demands on phonological encoding, storage, and speech‐output rather than tapping into familiar lexical access alone making it a purer measure to identify variations of PWM. Based on the published validity and reliability from the literature, ASRS is 6‐item DSM‐5 RiskSLIM‐weighted screener and shows high agreement with clinical diagnosis at the threshold ≥14 (sensitivity = 91.4%, specificity = 96.0%, and AUC = 0.94; Ustun et al. 2017), the SSI‐3 has good inter‐ and intra‐judge reliability with an overall reliability of approximately 87% for experienced clinicians (Riley 1994), and the UNWR shows excellent judge agreement for scoring (Howell et al. 2017).

### 2.4 Procedure

Participants were first assessed for vision and hearing. For the vision test, participants sat one meter away from the computer screen and indicated what direction of a progressively smaller stimulus (“*E*”) was facing with left eye covered. This procedure was then repeated for the right eye. The stated direction of the stimulus from the participant was inputted by the experimenter with a mouse click in the appropriate direction. Participants progressed in the study only when they had stated the correct direction of the stimulus for both eyes. For the hearing test, participants responded out loud if they heard an auditory stimulus that was played by the experimenter in the Optimus Nova 626 headphones at the following intensities (decibels, dB): 50, 45, 40, 35, 30, 25, 20, 15, 10, and 0; and at the following frequencies (kilohertz, kHz): 0.25, 0.5, 1, 2, 4, and 8. Participants were unaware of the intensity and frequency of the stimulus. They had to successfully detect the stimulus below 20 dB or at 20 dB per frequency to proceed.

Depending on the users ID, participants then either completed ASRS, SSI‐3, experiment and UNWR or first they completed UNWR, experiment, ASRS and then SSI‐3. In the ASRS questionnaire online users gave answers to six questions from never to very often. Then they read two short paragraphs from the SSI‐3 manual whilst they were video‐recorded (participants consented to being video‐recorded). UNWR was implemented as a web application^3^ and was based on the UNWR original version by Howell et al. (2017). ^4^ All the non‐word sounds were downloaded from the UNWR original version. UNWR consisted of four sets with 12 non‐words in total and two practice non‐words in each set. The experimenter first played the two practice non‐words to familiarize participants with the level and then they played the eight non‐words in each set. The web application version had a button that played the non‐words in a random order that the experimenter controlled. In UNWR, participants looked at the computer screen, heard the non‐words played over the Optimus Nova 626 headphones once they clicked a mouse, and then repeated out loud the non‐words. If the participant correctly attempted the non‐word, the experimenter continued with the next presentation. Otherwise, the task ended. If the participants repeated all eight non‐words correctly, they progressed to the next set (i.e., the set with one more syllable than they had heard so far). Accent differences were reduced by scoring consonants alone as in Howell et al. (2017).

### 2.5 Network Analysis

According to Epskamp et al. (2017a), the strength of the connection between two nodes in psychological NM networks is a metric determined from the data. Estimates are better for large size samples (power was >80% for this study). The relationships between different features were examined using network analysis, and the relevance of each feature was determined for each group (neurotypical participants, PWADHD and PWS). A pairwise Markov random field (PMRF) was used to generate the network model. PMRF estimates psychological networks and contains nodes that represent variables and edges without a directional arrow (Epskamp et al. 2017b). The size and density of the edges between the nodes represent the strength of connectedness. This indicates conditional dependence between features. Typically, NM involves two steps: (1) estimating a statistical model on data, from which some parameters can be represented as a weighted network between observed data, and (2) analysing the weighted NM using a graph to show the most central nodes. Centrality can be used to investigate the position of nodes in a network. Measures of centrality are strength, closeness and betweenness (see Section 2.6). The distinction between sparse and dense networks is crucial in terms of global topologies (Borsboom et al. 2021). In sparse networks a few edges are compared to the entire number of possible edges. In dense networks many edges are compared. This distinction is important for two reasons: (1) optimal estimation processes may be sparsity‐dependent; for example, regularization‐based approaches should perform well when data are generated from a sparse network, but not from dense networks; and (2) individual nodes are often more important in sparse networks than in dense networks, because all nodes in dense networks have a similar and large number of edges. In the following analysis, a sparse network (Epskamp et al. 2017b) was created, and a regularization parameter was used (LASSO—least absolute shrinkage and selection operator) for the correlation networks as it performs well in a sparse network and limits the number of spurious relationships (Hevey 2018). Although there are several LASSO approaches, the graphical LASSO (glasso, Friedman et al. 2007) is recommended for its ease of use in analysis. Extended Bayesian Information Criterion (EBIC; Chen and Chen 2008) was employed to choose the best NM, which is particularly effective when identifying the actual network structure (van Borkulo et al. 2014), especially when the true network is sparse. As a result, for the sparse network, the EBICglasso approach was adopted. The tuning parameter *λ* was set at 0.2 and the gamma γ was set to 0.2 (*γ* is typically set between 0 and 0.5; Foygel and Drton 2010). The higher the tuning parameter, the more edges from the network are deleted (Hevey 2018). A popular strategy for ensuring that the optimal tuning and gamma parameter is chosen is to estimate several networks with various values (Hevey 2018). For our networks no difference was observed in the NM for different tuning and gamma parameters. The NM graph was visualised with the R package qgraph developed by Epskamp et al. (2012). The networks were built using standardised diagnostic measures (ASRS, SSI‐3, and UNWR) because they assess common features of the conditions. The network demonstrates the relationships between the features. Positive and negative correlations (connections between nodes) are represented by green and red edges respectively. The degree of correlation is shown by the thickness of the edges. High ASRS suggests higher ADHD traits, high SSI‐3 suggests greater stuttering severity and high UNWR shows better PWM abilities.

Following the estimation of a network structure, the network graph's visualisation provides a thorough account of the multivariate relationships in the data. The estimate of PMRF is the state‐of‐the‐art method for determining undirected psychological network topologies. A PMRF is a network model in which edges represent conditional relationship between two nodes. The relationship between two nodes that results from their connection cannot be described by another network node. It may be explained simply as a correlation that controls all other relationships. Also, edges can show symmetric causal connections between features. All three networks were comparable for ASRS, SSI‐3 and UNWR across groups by applying an average layout in the statistical computing platform R.

### 2.6 Centrality Analysis

Centrality measures show which features have the strongest association with other features (Hevey 2018): The central nodes are more important than the peripheral nodes. Node centrality can be established using strength, betweenness and closeness measures obtained from the qgraph package in R (Epskamp et al. 2012) or the bootnet package (Epskamp et al. 2018). Strength indicates the most central feature. Centrality provides a measure of the absolute value of the edge weights connected to a node (Jones et al. 2019). Betweenness shows how many times/percentages a node is on the shortest path between two other nodes and can be used to predict which nodes are likely to function as connecting groups in a network. High betweenness nodes are important for connecting groups. Closeness estimates how close a node is to all other nodes on average in terms of the edge distance. This can be calculated by getting the average of the path from one node to every other node in the network. The node with the lowest average indicates that that node is closest to other nodes in the network. In psychological networks, strength has received the most attention, whereas betweenness and closeness centrality are less popular (Epskamp et al. 2018; Forbes et al. 2017).

The absolute value of every edge that connects SSI‐3 with ASRS and SSI‐3 with UNWR can be summed to understand the strength of SSI‐3. For example, if SSI‐3 with ASRS has an edge weight of ‐0.21 and SSI‐3 with UNWR has an edge weight of ‐0.43, the strength of SSI‐3 with ASRS and SSI‐3 with UNWR would be 0.21 + 0.43 = 0.64. For betweenness, the count or percentage of the number of times the SSI‐3 node is on the shortest path between ASRS and UNWR nodes is obtained. Betweenness is commonly used to understand whether one node plays a key role in connecting nodes with each other. For example, in the case where there are many nodes in a NM, some nodes can act as a bridge to connect some groups of nodes with other groups of nodes. In this study there was no key node that connected groups of nodes because SSI‐3, ASRS and UNWR are all inter‐connected. For the closeness of SSI‐3 to other nodes, the average distance between the SSI‐3 nodes with ASRS and UNWR is taken.

## 3 Results

Out of 179 participants, 177 were included in ASRS and UNWR. The two participants omitted for each measure were outliers. In SSI‐3, only 166 participants were included because data from 13 participants were missing due to equipment malfunction. Descriptive statistics showed that ASRS (Table 1 row 1) was highest in the PWADHD group, followed by PWS and neurotypical group indicating elevated ADHD traits in PWADHD. SSI‐3 (Table 1 row 2) was highest in PWS followed by PWADHD and neurotypical participants suggesting greater stuttering severity in PWS. Neurotypical group had the highest UNWR scores (Table 1 row 3) followed by PWADHD and PWS. These results indicate higher PWM performance in the neurotypical group compared with the other groups.

To assess subgroup overlap between PWS and PWADHD, cross‐trait analyses were conducted. Validated ASRS threshold (ASRS ≥ 14; Ustun et al. 2017) and conservative threshold based on the minimum score achieved in the clinically diagnosed group in PWADHD (ASRS ≥ 17) were utilised to assess variations in attention in PWS. Standard SSI‐3 bands (very mild: 10–17; mild: 18–24; moderate: 25–31; severe: 32–36; very severe: 37–46; Riley 1994) were utilised to assess stuttering‐like characteristics in PWADHD. Results showed that 17/79 participants had very mild stuttering characteristics (SSI‐3: 10–17), but only 1/79 reached mild score (SSI‐3 = 19), with none in the moderate or severe ranges. In PWS, 17/33 participants met the ASRS screening threshold (ASRS ≥ 14), and 5/33 the conservative ASRS threshold (ASRS ≥ 17). Results suggest that co‐occurring traits were present in the sample. However, the sample size was too limited to achieve stable network estimations.

**FIGURE 2:**
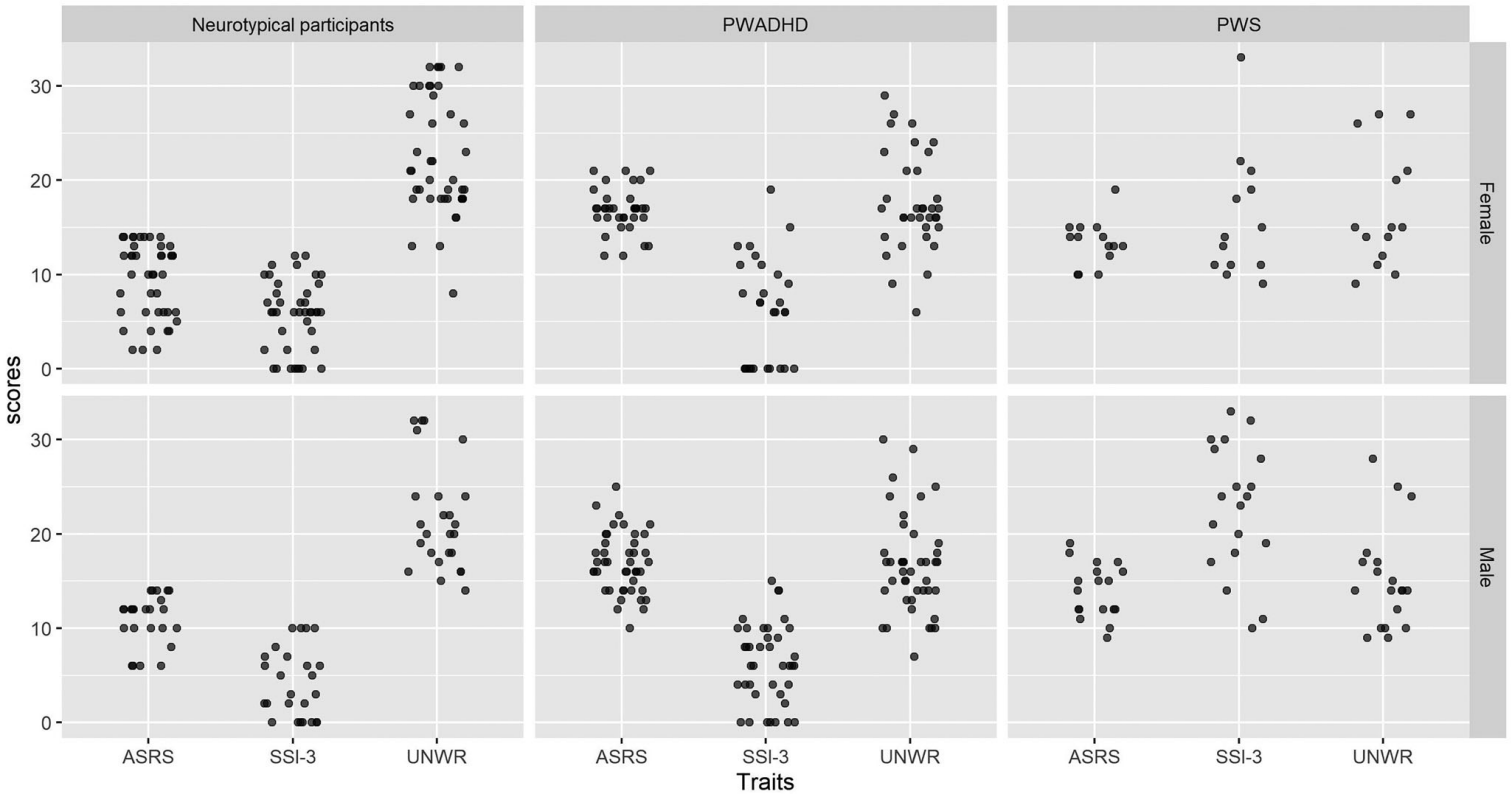
Ggplot conducted in R for the three dependent variables ASRS, SSI‐3 and UNWR in neurotypical participants, PWADHD, PWS for females (top row) and males (bottom row). Each feature is shown on the *x*‐axis and the y axis represents the scores for both genders.

A Linear Mixed Model, (LMM) was conducted to check whether gender difference between groups biased the three dependent variables ASRS, SSI‐3, UNWR (data in Figure 2). Two models were created. The first model looked at the effect of two fixed factors (1) measure type ASRS, SSI‐3 or UNWR; and (2) gender; and two random factors (participant and group). The second model looked at the effect of one fixed factor: measure type (ASRS, SSI‐3 or UNWR) and the two random factors (participant and group). Therefore, only model 1 included gender. The models were compared using ANOVA to determine whether removing the fixed factor gender significantly impacted the scores. No significant difference between the likelihood of the two models was observed (*χ* ^2^(1) = 0.201, *p* = 0.653). Hence, gender ratio differences between participant groups did not bias the scores.

**FIGURE 3:**
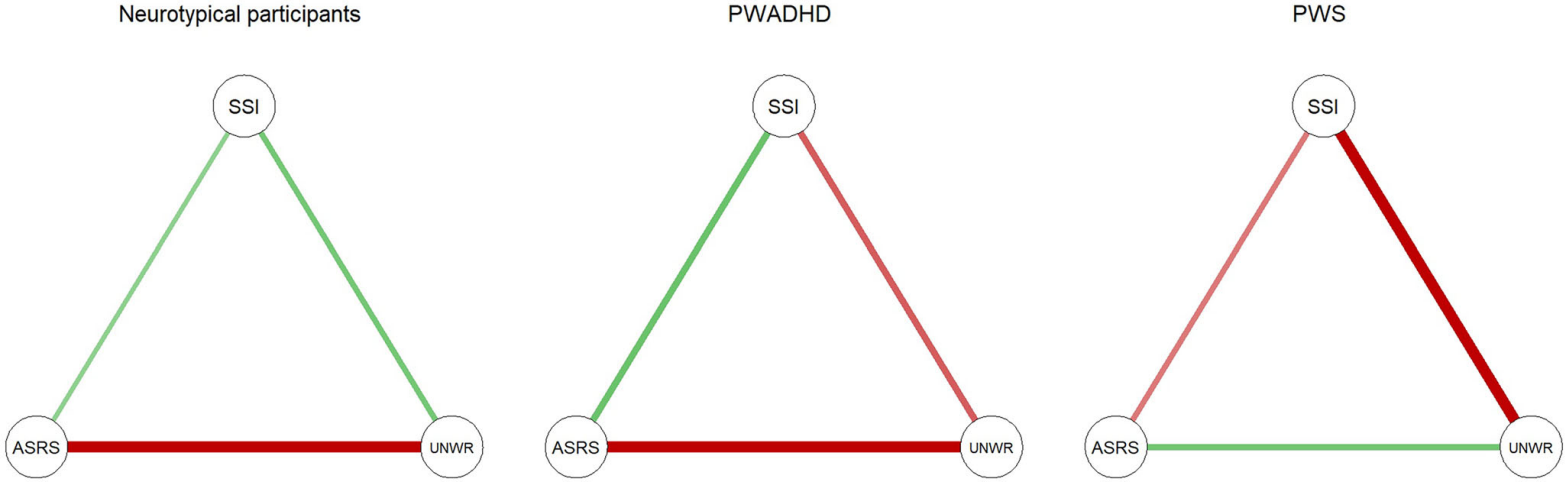
Network models for ASRS, SSI‐3 and UNWR in neurotypical participants, PWADHD and PWS. *Note*. Green and red connections between nodes represent positive and negative correlations respectively. A positive sign means that nodes covary in the same direction, whereas a negative sign means they covary in the opposite way. The degree of correlation is shown by the thickness of the connections. The green line between ASRS and SSI‐3 in the middle NM, indicates PWADHD who had high ASRS scores showed greater stuttering characteristics, but this was opposite for PWS as shown in the third NM. PWS who have high ASRS scores (elevated ADHD traits) showed lower stuttering characteristics.

Figure 3 shows that NMs differed between groups supporting H1. NM for neurotypical participants showed the link between ASRS (attention) and UNWR (PWM) was important but neither of these related to the SSI‐3 (fluency) measure. The direction was that when ASRS scores were high (poorer attention) UNWR scores were lower with a correlation of −0.34 for neurotypical participants (Table 2) supporting H2. Positive weak partial correlations occurred between SSI‐3 and ASRS (greater stuttering severity with elevated ADHD traits) and between SSI‐3 and UNWR (greater stuttering severity with higher PWM) with coefficients 0.15 and 0.18 respectively as shown in Table 2.

**TABLE 2:** Partial correlations coefficients for neurotypical participants, PWADHD and PWS for each pair of scores.

	SSI‐3‐UNWR	ASRS‐UNWR	ASRS‐SSI‐3
Neurotypical participants	0.18	−0.34	0.15
PWADHD	−0.16	−0.25	0.14
PWS	−0.21	0.12	−0.12

*Note*: All coefficients are partial correlations retrieved from the network models reported in Figure 3.

PWADHD showed high ASRS scores (elevated ADHD traits), and this correlated with higher SSI‐3 scores (greater stuttering severity; *r* = 0.14) and with low UNWR scores (lower PWM performance; *r* = −0.25). High UNWR scores led to low SSI‐3 scores (reduced stuttering severity; *r* = −0.16) as well as low ASRS scores (lower ADHD traits), as mentioned.

PWS lost the link between ASRS and UNWR scores suggesting that having good attention abilities did not help PWM (*r* = 0.12). PWS who have high UNWR scores (higher PWM performance) had low SSI‐3 scores (better fluency) which correlated −0.21 (similar to PWADHD). There was a lesser indication that high SSI‐3 scores (low fluency) were associated with high ASRS scores (elevated ADHD traits) where the correlation was −0.12 in the direction expected. With respect to H3 the link between UNWR and ASRS for PWADHD was similar to neurotypical participants but for PWS this pattern was lost.

Examination of the networks in Figure 3 and correlation matrices in Table 2 shows SSI‐3 (fluency) was not important in neurotypical group but there was a strong negative link between ADHD measure (ASRS) and UNWR performance. Attention impacts PWM which affects many cognitive functions including speech production. In PWS, the variation in attention ability did not covary with PWM in the same direction as in the other groups. To understand whether this is attributable to a ceiling effect, the means of all groups for all measures are given in Table 1. This confirms that there is no ceiling effect for ASRS (maximum score of 25), SSI‐3 (maximum score 46) and UNWR (maximum score of 32).

**FIGURE 4:**
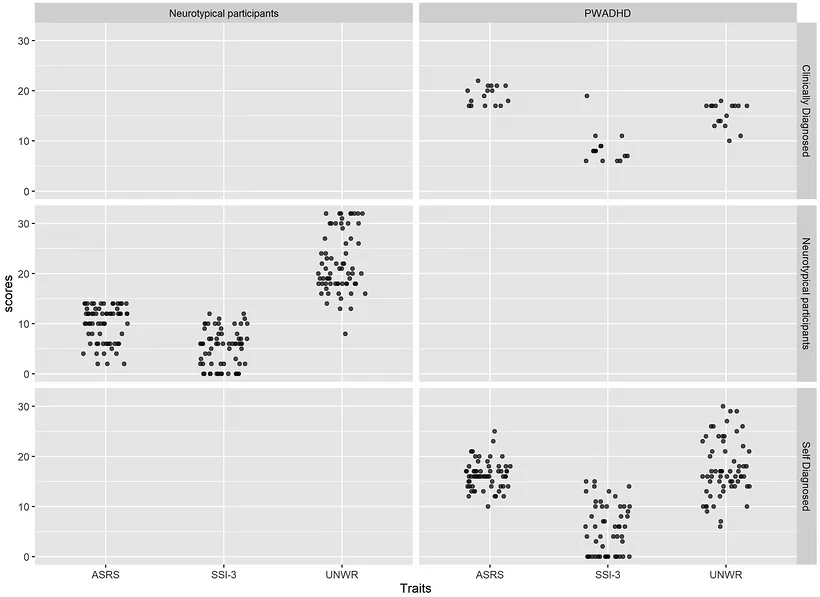
Ggplot for ASRS, SSI‐3 and UNWR in neurotypical participants, PWADHD self‐diagnosed and PWADHD clinically diagnosed. Features are shown on the *x*‐axis and the y axis represents the scores for groups.

To determine whether NM architecture was similar between neurotypical participants and self‐diagnosed PWADHD in comparison to neurotypical participants and clinically diagnosed group, NMs and LMM analysis were conducted. Figure 4 displays ASRS, SSI‐3, and UNWR scores across neurotypical participants, self‐diagnosed PWADHD, and clinically diagnosed PWADHD. No difference was seen between PWADHD and neurotypical group for both ADHD subtypes according to visualizations from NMs. Two models were created in LMM, one that included subtypes of ADHD group and one without subtypes. The two models were compared with ANOVA to determine whether subtypes differed. No significant difference was observed (*χ* ^2^(2) = 5.234, *p* = 0.073) between the two models. Hence, subtypes did not affect the scores.

**FIGURE 5:**
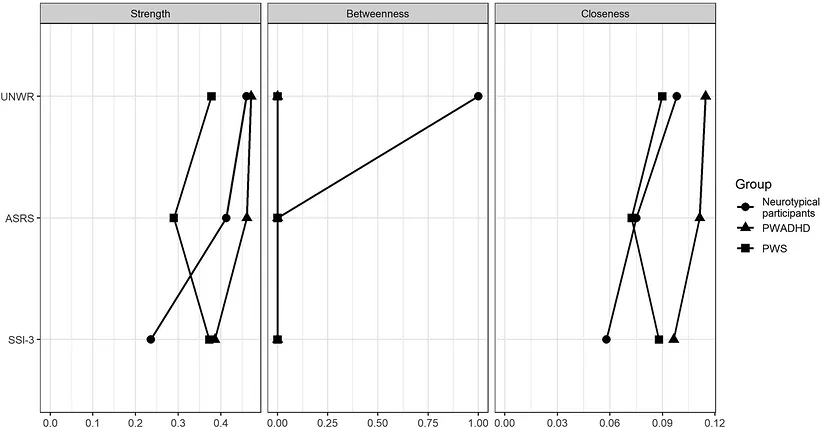
Centrality of features (ASRS, SSI‐3 and UNWR) in neurotypical participants, PWADHD, and PWS ordered by strength. *Note*: UNWR has the highest strength (shown by the left subfigure) in each group as also determined by having the highest score (*x* axis). ASRS is not shown in betweenness (middle subfigure) because it's score is 0, suggesting that none of the measures in PWADHD is important in connecting other nodes.

Figure 5 shows that in neurotypical participants, PWS and PWADHD, UNWR is the most central factor as observed from strength. This suggests that PWM is important and has strong connections to other nodes. With respect to betweenness in neurotypical group, only UNWR has importance in acting as a bridge to other nodes. UNWR has the highest closeness in neurotypical participants, PWS and PWADHD, suggesting PWM is an important factor in all groups.

Network comparison tests, (NCTs) were conducted to assess the differences in connectivity between participant groups for: (1) edge differences; and (2) node centrality. These were not statistically significant for any of the three measures tested (ASRS, SSI‐3, UNWR). Network accuracy and stability were assessed using the bootnet package (Epskamp et al. 2017a), using nonparametric bootstrapping of edge weights (2000 resamples, 95% CIs) and case‐dropping subset bootstrapping for centrality (CS‐coefficient). The most robust and accurately estimated edges were negative ASRS‐UNWR associations in both the neurotypical group (edge = −0.34, 95% CI [−0.57, −0.10]) and ADHD group (edge = −0.24, 95% CI [−0.45, −0.04]; see Table 2). As expected from a network model with three nodes, the centrality indices (closeness and betweenness) showed limited stability (CS < 0.25). Accordingly, the main interpretations will focus on the most accurately estimated edge‐level associations, while centrality comparisons and differences involving the PWS network are treated as exploratory.

## 4 Discussion

### 4.1 Summary

Network analysis in this present study, provides insights on the associations among attention (ASRS), fluency (SSI‐3), and PWM (UNWR) across neurotypical participants, PWADHD, and PWS. Findings contribute to our understanding of the complex interplay between cognitive domains and speech production, offering novel perspectives on the underlying mechanisms that differentiate studied neurodevelopmental profiles.

Descriptive statistics revealed the expected group profiles. ASRS scores were highest in the ADHD group indicating higher variations in attention, SSI‐3 scores were highest in the stuttering group showing higher stuttering characteristics in PWS and PWM abilities were highest in the neurotypical group as observed from higher UNWR scores. Differences from all three groups were large, indicating that the sample could reliably detect the effects present in the data. It was hypothesised that attention, fluency and PWM operate differently between neurotypical participants, PWS and PWADHD. In terms of connectivity patterns, NMs differed between participant groups supporting H1. PWM was related to attention in neurotypical group as expected with higher UNWR scores (better PWM) linked to lower ASRS scores (better attention) supporting H2. This pattern was similar for PWADHD but differed for PWS suggesting that PWM ability may not have the same effect on attention in this group of participants supporting H3. Although PWM is a strong factor in all groups having the highest strength and closeness as assessed by centrality analyses, the way it operates between PWADHD and PWS differs. Different patterns of association of PWM with attention and fluency across groups were displayed, suggesting that similar surface‐level trait profiles do not imply shared cognitive architecture. Findings argue against a shared underlying mechanism from trait overlap alone and instead they support a dimensional, network‐based understanding of neurodevelopmental profiles. Although, NCTs did not reach significance, the limited power of NCT with three‐node networks and sample size (van Borkulo et al., 2022) is discussed below. Our results are consistent with the DSM‐5 conceptualisation of both ADHD and stuttering as neurodevelopmental disorders, characterised by early onset, and continuation across development, and heterogeneous cognitive profiles (American Psychiatric Association 2022). Although PWS and PWADHD may report overlapping traits, DSM‐5 does not conceptualise these conditions as sharing a single underlying mechanism. In contrast, neurodevelopmental disorders can present overlapping trait dimensions that arise from distinct developmental pathways.

Several control analyses ruled out extraneous variables affecting the results. For instance, gender ratio differences across participant groups (Section 2.1.) did not affect ASRS, SSI‐3, or UNWR scores. Likewise, the different ways ADHD was diagnosed (Section 1.3.) did not alter the model fit suggesting that the findings were not driven by ADHD diagnostic subtype. Mean scores for all the measures were well below their maximum score, indicating that the difference of the ASRS‐UNWR association in PWS compared to other groups was unlikely to have been caused by ceiling effects.

Network accuracy analyses showed that the negative ASRS‐UNWR associations in both neurotypical and ADHD groups were among the most accurately estimated in the networks. As expected for three‐node networks, centrality stability was limited (CS < 0.25). NCT showed no significant differences in edge weights or node centrality between any of the three groups (neurotypical participants, PWS, PWADHD) suggesting similar overall connectivity and strength of associations among ASRS, UNWR, and SSI‐3. However, NCTs assesses global network invariance rather than individual edge differences (van Borkulo et al. 2022). In the present study, a non‐significant difference between the NMs cannot be interpreted as the networks were identical since the results may reflect a limited power to detect differences that could arise from small number of nodes and unequal sample sizes compared to those validated in van Borkulo et al. (2022). Hence, two interpretations of the group‐specific edge patterns are considered below.

On one account, the asymmetric edge patterns may reflect different organisations of attention, fluency, and PWM across groups. ADHD has been characterized as a disorder of self‐regulation and executive control resulting in inconsistent performance rather than a reorganisation of underlying cognitive structure (Barkley 2011). As a result, the overall structure of cognitive relationships in PWADHD resembles that of neurotypical group. A higher phonological complexity in UNWR, may place greater demands on attentional control and executive‐loop maintenance in PWADHD in producing accurate non‐word repetitions. In contrast, stuttering has been linked to difficulties in timing and coordination between planning and execution rather than differences in a single speech component (Howell 2004; Howell et al. 1999; Max et al. 2004). This might explain why PWM in PWS does not support attention in the same way as in neurotypical participants but instead appears to support speech fluency leading to a possible reorganisation of network structure. Consistent with Pennington's (2006) framework, findings suggest that trait overlap does not indicate shared underlying mechanisms. In the present study, attentional difficulties in PWADHD occur within an unchanged network architecture (e.g., sometimes fail to self‐regulate so attention is inconsistent) reflecting variability in self‐regulation rather than structural reorganization. In PWS, attentional difficulties emerge secondarily within a different pattern of associations among features (e.g., the network has been reorganised so attention can support speech not because attention is the main feature). Consistent with the EXPLAN model (Howell 2004) which proposes that stuttering in PWS reflects a temporal asynchrony between independent linguistic planning and motor execution, the strong association of fluency and PWM in PWS may indicate greater reliance on maintaining and coordinating phonological plans. These differences may explain the asymmetric patterns relative to neurotypical participants and indicate partially distinct cognitive mechanism in PWS and PWADHD.

Alternatively, we cannot exclude the possibility that unscreened co‐occurring neurodevelopmental profiles such as dyslexia may have contributed to the slightly weaker patterns, particularly SSI‐3‐UNWR association in PWADHD relative to PWS. Studies report that dyslexia co‐occur frequently in children with either reading disability or ADHD (25%–40%; McGrath et al. 2010; Willcutt et al. 2010). In their study, Elsherif et al. (2021) reported that 50% of AWS (*N* = 30) fulfilled the criteria for the dyslexia diagnosis and 17 out of 50 (34%) of adults with dyslexia (AWD) reported stuttering. Furthermore, they found a similar lower PWM, awareness and retrieval in AWS and AWD compared to neurotypical participants suggesting a shared phonological trait across both profiles. This might suggest that the weaker association of SSI‐3‐UNWR in PWADHD may reflect underlying variability in reading and phonological variation that could increase influence on storage and retrieval demands to UNWR performance and weaken the link with SSI‐3. In a recent study, Elsherif et al. (2025) assessed AWS, and AWD in a Stroop task and compared them to neurotypical participants. Results showed that both AWS and AWD showed elevated Stroop interference which was moderated by lexical precision. Participants with a higher lexical precision exhibited Stroop effects closer to neurotypical participants. Importantly, AWS and AWD were similarly affected on the task, suggesting that lexical representations may affect cognitive control. Elsherif et al. (2026) assessed AWS, AWD, and neurotypical participants on phonemic, semantic, and design fluency tasks. Hierarchical regression showed that both dyslexia and stuttering predicted lower phonemic fluency output and fewer subcategory switches, but neither predicted semantic or design fluency scores. Stuttering additionally predicted smaller phonemic clusters. These results suggest that despite the similarity in the overall phonological pattern in dyslexia and stuttering, their underlying phonological mechanisms are distinct. This distinction is relevant here because despite that UNWR taps into PWM, a lower performance might also reflect underlying processes such as phonological representation, retrieval efficiency, or speech‐related planning mechanisms. Therefore, similar associations between PWM and other measures may not necessarily reflect a shared architecture. Applied to the present networks, findings from dyslexia and stuttering research may indicate that unscreened dyslexic traits have contributed to slightly weaker SSI‐3‐UNWR association in PWADHD shifting UNWR performance toward phonological storage and retrieval demands rather than speech‐motor fluency. Hence, findings may indicate that the weaker association may partly reflect unscreened dyslexic traits influencing UNWR performance, rather than a separate ADHD cognitive organisation in which PWM is linked to attention but not to fluency. However, this possibility may not fully account for the preserved ASRS‐UNWR link in neurotypical participants and PWADHD but not PWS, and the opposite ASRS‐SSI‐3 directions which remain more suggestive of partially distinct cognitive architectures.

### 4.2 Theoretical and Clinical Implications

The main theoretical implication of this study is that similar behavioural profiles do not necessarily imply a shared cognitive organisation. Both PWS and PWADHD showed elevated traits associated to the other condition but NMs differed in how attention, fluency and PWM were associated. In particular, the association ASRS‐UNWR observed in both PWADHD and neurotypical participants was not observed in PWS. Additionally, SSI‐3‐UNWR link was stronger in PWS. Findings suggest that trait overlap can arise from distinct neurodevelopmental pathways in line with the dimensional view of neurodevelopmental disorders rather than reflecting a common latent cause (American Psychiatric Association, 2022; Pennington 2006). One possible interpretation is that PWADHD resembled neurotypical participants in the overall organisation of the three measures assessed here, while PWS may show a reorganised architecture. This does not imply that attention is irrelevant in PWS. Instead, it suggests that attention and PWM were not associated in the same way. Clinically, findings suggest that overlapping traits of attention, fluency and PWM in PWS and PWADHD should not be automatically interpreted as evidence of a shared profile. Assessments should also consider how these measures relate within a person or a group whose primary condition is either stuttering or ADHD instead of only assessing whether elevations exist. Results also highlight the important role that PWM has in these conditions supporting the inclusion of PWM assessment, particularly cross‐linguistic NWR tools such as UNWR (Howell et al. 2017) which incorporates greater demands of phonological information and higher quality of phonological representations to reveal significant group differences (Gerwin et al. 2022; Pelczarski and Yaruss 2016; Yang et al. 2019). Network approaches offer a useful way to distinguish surface trait overlap from differences in cognitive organisation. NMs also provide excellent visualisations and have showed to be particularly useful in understanding communication processes in stuttering research (Castro et al. 2017) thereby supporting more profile‐specific clinical assessments and treatments.

### 4.3 Limitations and Constraints on Generality

In line with contemporary reporting standards for constraints on generality (Simons et al. 2017), the following limitations bound the populations to which the present network findings generalize. The present findings are most directly applicable to adults who speak English and are classified as neurotypical or who have a primary condition of ADHD or stuttering assessed in ASRS (Ustun et al. 2017), SSI‐3 (Riley 1994), and UNWR (Howell et al. 2017) in three‐node NMs of attention, fluency and PWM measures. We expect the edge‐level patterns to be most applicable to comparable samples recruited through university pools, online ADHD forums, and stuttering self‐help groups. We do not assume direct generalizability to children, to samples with formal screening for co‐occurring dyslexia or another neurodevelopmental profiles. We also do not assume generalizability to other attention, fluency or PWM metrics, or to cross‐linguistic and culturally diverse cohorts.

Several limitations restrict the generality of the present findings. First, the sample sizes were unequal across neurotypical participants (*N* = 67), PWADHD (*N* = 79) and PWS (*N* = 33). Sensitivity analyses indicated that the study could reliably detect large‐group differences observed on ASRS, SSI‐3, and UNWR whereas smaller effects from pairwise comparisons involving PWS may not have been detected limiting the power of global NCT. Second, the networks comprised three nodes. This design was appropriate as a first study that assesses attention, fluency and PWM through NMs, but it may have simplified more complex cognitive systems associated with both conditions. The most robust associations were observed in the neurotypical and PWADHD networks, and therefore the main conclusions are drawn from these associations, with patterns in PWS and centrality indices interpreted more cautiously. Third, participants were grouped by primary condition which allowed clear comparisons of NMs across groups. Co‐occurring profiles were not screened in this study as it was not a criterion and thus NM differences across subgroups were not determined. Instead, overlapping traits were modelled continuously within each network. Further analysis at screening level confirmed limited cross‐trait overlap. Therefore, the subgroup sample was too small to conduct stable network analysis. However, latent co‐occurring traits may still have contributed to the observed edge associations within the PWS and PWADHD networks. Fourth, higher rates of dyslexia have been reported in both stuttering and ADHD (Elsherif et al. 2021; Elsherif et al. 2025; McGrath et al. 2010; Willcutt et al. 2010) and unscreened related dyslexic traits as well as related phonological traits such as phonological storage traits may have affected how PWM related to fluency, limiting conclusive interpretations in UNWR‐related edges and in particular to the slightly weaker SSI‐3‐UNWR association in PWADHD. Fifth, constraints associated with the UNWR task utilised in the present study should be acknowledged. Although UNWR shows excellent judge agreement for scoring performance, UNWR is based on a validated total accuracy score and applies a discontinued rule. Hence, item‐level error‐type information were not applicable. Consequently, the networks reflect overall PWM performance rather than distinctions between storage, retrieval, or speech‐motor breakdown limiting interpretations to further edge‐associations. Additionally, shorter versus longer nonwords or tasks that place greater demands on passive storage versus cognitive‐motor assembly may have affected edge associations. Furthermore, while UNWR has been designed to reduce lexical bias and is a validated PWM measure with lower neighbourhood density, NWR tasks remain influenced to some degree by vocabulary, phonological knowledge, and processing strategy. The present networks therefore may have not fully isolated PWM from all lexical processes. As outlined above, item‐level error‐type information of PWM was not applicable in the present study and inclusion of such data would allow for more refined interpretation of how PWM relates to attention and fluency. Work currently under revision (Howell et al., in revision) applied transcription‐based substitution, deletion and insertion (SDI) scoring of UNWR task (Howell et al. 2017), together with perceptual‐saliency (PSal) measures, to examine how these metrics distinguished neurotypical participants from PWS and PWADHD. Findings suggested that model fit for distinguishing PWS from neurotypical participants was significantly improved when both SDI and PSal parameters were combined compared to using SDI or PSal alone. It was also concluded that substitute errors in PWS were avoided when salience properties of UNWR stimuli were higher. Whereas in PWADHD no such effects were observed indicating that the lower levels of attention might have limited their ability to avoid substitution errors. Findings also support the view that item‐level linguistic and sensory properties can contribute differently across groups. Hence, the UNWR scores may therefore reflect the overall PWM performance, instead of a pure measure of short‐term phonological storage independent of vocabulary, reading experience, or lexical knowledge. Sixth, participants were recruited tested in English, and drawn from university pools, online ADHD forums, and stuttering self‐help groups. Participants were recruited in accordance with the university's research ethics procedures which required participants to have conversational or fluent proficiency in English. PWADHD group comprised of clinically diagnosed and non‐clinical cases, and socio‐demographic, dialect, and print‐exposure variables were not fully characterised. Executive function, vocabulary, and speech‐motor style may differ according to socio‐linguistic background, and therefore the observed NM findings should be considered as specific to this sample and not representative of diverse cohorts of stuttering or ADHD. Finally, individual variations of attention, fluency and PWM across groups may have not been captured since NMs in the present study represent group association patterns. Hence, group‐level edges may mask subgroups with different cognitive organisations limiting the extent to which these findings can be generalised to the individual level.

### 4.4 Future Directions

Future studies should extend these findings in several ways. First, larger and more balanced samples will improve the stability of network estimates and support more robust conclusions about the cognitive architectures of PWS and PWADHD. Second, assessing co‐occurring subgroups in sufficiently large samples will help clarify how fluency, attention, and PWM are associated when these profiles overlap. Third, measures that assess dyslexia traits should be included so that networks can capture the influence of these traits on attention, fluency and PWM. Fourth, future studies should take into consideration SDI and PSal parameters alongside composite UNWR scores when measuring PWM, as well as incorporating NWR tasks with greater phonological complexity to determine whether group differences are due to storage, retrieval, executive, speech‐motor, lexical‐phonological, or sensory‐perceptual processes. Fifth, instead of using SSI‐3 to measure fluency, a case can be made for just investigating the percentage of stuttered syllables (%SS) component of SSI‐3 (Mirawdeli and Howell 2016). Also, in terms of fluency measure, SSI‐3 excludes whole‐word repetitions which are associated with hesitancy in speech. A case can be made for examining %SS and a similar measure of hesitancy in speech. Using %SS of SSI‐3 would provide a less noisy measure of stuttered speech for all groups and inclusion of a hesitancy measure might help characterize fluency characteristics more adequately for typical speakers. Similarly extending attentional measures in other ways (e.g. executive functioning) may provide a more refined measure for PWADHD and highlight differences relative to the other participant groups. Finally, examining networks at an individual level may help determine whether the patterns observed in PWS and PWADHD apply across individuals or whether different subgroups with different cognitive profiles emerge. This approach may enhance the clinical utility of NMs for understanding whether different underlying mechanisms involve similar traits across neurodevelopmental profiles.

### 4.5 Conclusions

To conclude, the patterns of association among attention, fluency and PWM differed across groups. PWM was central in all networks but the way it affected attention and fluency was not the same in PWS, PWADHD and neurotypical participants. Findings showed that overlapping traits do not necessarily suggest a shared underlying cognitive mechanism. Instead PWS and PWADHD show different organisations among the measures assessed. Furthermore, the way the three features relate to one another in PWADHD is similar to the neurotypical group, but both differ from PWS. Study supports NM as a useful approach to understand co‐occurrence and underlying mechanisms across neurodevelopmental profiles provided that findings are interpreted within the constraints of the sample, measures, and co‐occurring profiles discussed here.

## Ethics Statement

The study was approved by University College London, (UCL) Research Ethics Committee (6252/002). Participants read an information sheet and signed a consent form prior to conducting the study.

## Data Availability

The datasets generated and analysed for this study can be found in the paper's Open Science Framework Page: [https://osf.io/mne2u/overview?view_only=c3343315d5ef48789bf7cdea7a81d3ca] (this is a reviewers' only version; the repository will be made public upon acceptance).

## Endnotes

1 We use neurodiversity‐affirming language when describing our own findings (e.g., co‐occurrence, traits, features, variations, profiles, fluency/stuttering characteristics, and stuttering‐like characteristics). Terms such as disorder, comorbidity, and symptom are retained only for DSM‐5 definitions, validated instruments (ASRS; SSI‐3), or cited literature.

2 Information about the NCS is available at https://rckessler.scholars.harvard.edu/ncs
